# Effect of *folA* gene in human breast milk-derived *Limosilactobacillus reuteri* on its folate biosynthesis

**DOI:** 10.3389/fmicb.2024.1402654

**Published:** 2024-05-15

**Authors:** Yu Jiang, Xianping Li, Wei Zhang, Yadong Ji, Kai Yang, Lu Liu, Minghui Zhang, Weicang Qiao, Junying Zhao, Mengjing Du, Xiaofei Fan, Xingfen Dang, Huo Chen, Tiemin Jiang, Lijun Chen

**Affiliations:** ^1^South Asia Branch of National Engineering Center of Dairy for Maternal and Child Health, Guilin University of Technology, Guilin, China; ^2^National Engineering Research Center of Dairy Health for Maternal and Child, Beijing Sanyuan Foods Co., Ltd., Beijing, China; ^3^Beijing Engineering Research Center of Dairy, Beijing Technical Innovation Center of Human Milk Research, Beijing Sanyuan Foods Co., Ltd., Beijing, China

**Keywords:** *Limosilactobacillus reuteri*, folate, molecular breeding, metabolism, transcription, probiotic properties

## Abstract

**Introduction:**

Folate supplementation is crucial for the human body, and the chemically synthesized folic acid might have undesirable side effects. The use of molecular breeding methods to modify the genes related to the biosynthesis of folate by probiotics to increase folate production is currently a focus of research.

**Methods:**

In this study, the folate-producing strain of *Limosilactobacillus reuteri* B1-28 was isolated from human breast milk, and the difference between B1-28 and *folA* gene deletion strain *ΔFolA* was investigated by phenotyping, *in vitro* probiotic evaluation, metabolism and transcriptome analysis.

**Results:**

The results showed that the folate producted by the *ΔFolA* was 2–3 folds that of the B1-28. Scanning electron microscope showed that *ΔFolA* had rougher surface, and the acid-producing capacity (*p* = 0.0008) and adhesion properties (*p* = 0.0096) were significantly enhanced than B1-28. Transcriptomic analysis revealed that differentially expressed genes were mainly involved in three pathways, among which the biosynthesis of ribosome and aminoacyl-tRNA occurred in the key metabolic pathways. Metabolomics analysis showed that *folA* affected 5 metabolic pathways, involving 89 different metabolites.

**Discussion:**

In conclusion, the editing of a key gene of *folA* in folate biosynthesis pathway provides a feasible pathway to improve folate biosynthesis in breast milk-derived probiotics.

## 1 Introduction

Folate (folic acid, FA) is a water-soluble vitamin that is absorbed in the body in active and passive ways, with absorption sites mainly in the upper part of the small intestine (Farrell et al., 2023). The absorbed FA is stored in tissues such as the intestinal wall, liver, and bone marrow, where it is reduced to physiologically active tetrahydrofolate by folate reductase with the combination of nicotinamide adenine dinucleotide phosphate (NADPH). The resulting tetrahydrofolate participates in the synthesis of purines and pyrimidines and is necessary for DNA synthesis and epigenetic regulation (Engevik et al., 2019). During embryonic development, a lack of active FA may lead to various neonatal birth defects such as neural tube defects (Ferrazzi et al., 2020), congenital heart disease (Chen et al., 2022), infant cognitive impairment (Pourié et al., 2020), and many other conditions. It has also been shown that FA may alter the methylation profile of JAK–STAT and long-term depression signaling pathways in Alzheimer’s disease models (Li et al., 2016). Folate-containing alginate microbeads has enhanced anti-tumor efficacy in targeted treatment of colorectal cancer (Rajpoot and Jain, 2020).

Folates cannot be endogenously synthesized in mammals folates, so its supply relies on dietary and supplemental intake (Clare et al., 2019). The recommended intake of folates for adults is 200–400 μg per day, while that for pregnant women is 400–600 μg per day (Tamene et al., 2022). Excessive intake of FA may affect the absorption of vitamin B12 (Girard and Duplessis, 2023), increase the risk of colon cancer (Kok et al., 2020), and impair the secretion of insulin so that hepatic fat metabolism is compromised (Kintaka et al., 2020). The current production of folic acid is mainly based on chemical synthesis, and the use of synthetic folic acid may cause many additional side effects (Field and Stover, 2018). It has been reported that FA biofortified yoghurt can regulate intestinal dysbiosis in folate-deficient rats, whereas chemical FA exacerbates intestinal dysbiosis (Zhang et al., 2020a). In contrast, these issues have not been reported on the natural folate obtained from probiotics through microbial fortification processes (Field and Stover, 2018). This is because it not only increases folate content but also exerts the probiotic function of probiotics (Rossi et al., 2011). Human breast milk is not only the healthiest source of nutrients for infants (Verhasselt, 2010) but also an important source of flora for infant growth and development. *Lactobacillus* and *Bifidobacterium* isolated from human breast milk are beneficial to infant health (Wu et al., 2021). Human breast milk-derived probiotics can promote the development of immune system (Sánchez et al., 2021), enhance nutrient metabolism and absorption (Bode, 2012), improve intestinal barrier function (Lyons et al., 2020), and stimulate the gut-brain axis (Palmer, 2011).

Folate produced by microorganisms has no side effects in the human body (Jiao et al., 2020), but the resulting folate content is relatively low. However, microbial molecular breeding can solve the above problems by transforming strains for specific production purposes to obtain high-yield strains, thereby meeting specific needs. So far, limited studies have been conducted to improve FA yield in human breast milk-derived probiotics through molecular breeding techniquest. In this study, the *folA* gene, encoding the dihydrofolate reductase in human breast milk-derived *Limosilactobacillus reuteri* B1-28 (B1-28), was knocked out by gene editing, resulting in 2-fold to 3-fold increase of folate production in the *ΔFolA* strain than that of the wild-strain, and the probiotic characteristics of B1-28 and *ΔFolA* were evaluated, including three-dimensional morphological structure, growth curve, hemolytic activity, resistance to acid, bile salt and antibiotics, adhesion to human colon cancer cell and resistance to pathogenic bacteria. The results of *in vitro* experiments showed significant differences between B1-28 and *ΔFolA*. In addition, metabolomic and transcriptomic differences between human breast milk-derived strains B1-28 and *ΔFolA* were further studied.

## 2 Materials and methods

### 2.1 Bacterial strains, culture conditions, and media

The probiotic strains involved in this study were provided by the Lactobacillus Bank of National Engineering Research Center of Dairy Health for Maternal and Child of Beijing Sanyuan Foods Co., Ltd. *Escherichia coli* (ATCC 25922), *Staphylococcus aureus* (ATCC 25923), *Salmonella enteritidis* (ATCC 14028), *Bacillus cereus* (ATCC 14579), *Listeria monocytogenes* (ATCC 19115), and *Salmonella typhimurium* (CMCCB 50071) were purchased from Guangdong Microbial Culture Collection Centre. All probiotic strains were cultured in MRS broth (Beijing Land Bridge Technology Co., Ltd.) at 37°C, and pathogenic bacteria were cultured in Luria-Bertani medium (Beijing Land Bridge Technology Co., Ltd.) at 37°C.

### 2.2 Screening of human breast milk-derived strains with high folic acid yield

Bromocresol violet, an indicator, was added into folic acid casei medium (FACM, Difco) at pH 6.8, and the activated strains was cultured at 37°C for 1–3 days. FACM is a medium for the determination of folic acid, consisting of 10 g/L acid hydrolyzed casein (without vitamins), 40 g/L glucose, 40 g/L sodium acetate, 1 g/L dipotassium phosphate, 1 g/L potassium dihydrogen phosphate, etc. If the medium solution turns yellow, it indicates positive, indicating that the strain is capable of synthesizing FA (Laiño et al., 2012). The FA content of the samples was detected according to the method described in the previously published literature (Qiao et al., 2022), Aluminum foil was used to avoid exposing the samples to light for all the following steps. The pH of 4 mL samples was adjusted to 1.7 with 1 M HCl then kept in the dark for 2 min. The pH was then adjusted to 4.7 using 1 M NaOH and placed in the dark for 2 min. After making up to 10 g with water, the samples were centrifuged and defatted at 10000 × *g* for 20 min 4°C (Sigma, Gottingen, Germany). Finally, the supernatants were removed and filtered through a 0.22-μm syringe filter. The samples were analyzed by an UltiMate 3000 (Accela)-Q Exactive mass spectrometer (Thermo Fisher Scientific, Waltham, MA, USA) with an ACQUITY UPLC HSS T3 column (HSS T3, 50 mm × 2.1 mm i.d., 1.8 μm) being used for separation. The MS conditions were as follows: source temperature 110°C, desolvation temperature 350°C, desolvation gas flow (N2) 700 L h^−1^, cone gas flow 30 L h^−1^, and collision gas flow (Ar) 0.13 mL min^−1^. Thermo Scientific Xcalibur software was used for system control and data management. Three parallel samples in each group.

### 2.3 The *folA* gene knockout in human breast milk-derived *Limosilactobacillus reuteri* B1-28

In this study, *L. reuteri* B1-28 was selected to construct a knockout vector using a gene editing system. Primers and upstream and downstream homology arms were designed according to the target gene *folA*, and the knockout vector was constructed accordingly. A recombinant plasmid was constructed using a temperature-sensitive plasmid, pk18mobsacB (Harighi, 2009; Wang et al., 2018), and then introduced into the target strains for the clone screening. The knockout strain was obtained by electroporation transformation (Wang et al., 2018; Yamamoto et al., 2018; Yao et al., 2021). The transformed *L. reuteri* B1-28 were sequentially screened in an LB medium containing 50 μg/mL kanamycin to remove the target gene, and then in an MRS solid medium containing 10% sucrose to remove the plasmid. The resulting knockout strain was named *ΔFolA*.

### 2.4 Determination of pH and FA content before and after fermentation

The activated B1-28 and *ΔFolA* were passed to the third generation, then the bacterial solutions were diluted to 10^8^ CFU/mL with sterile PBS. They were inoculated into sterilized MRS broth and cow’s milk at 6%, respectively, and then cultured at 37°C for 30–35 h. The pH values of the culture medium were measured using a pH meter (Five Easy Plus, Mettler Toledo Instruments (Shanghai) Co., Ltd., Shanghai, China) before and after fermentation. The pH meter is calibrated prior to use, the electrode is placed in the standard solution sample, the measurement button is pressed, the electrode is held in the solution for 1–2 min to ensure that an accurate reading can be made, and the pH level is set once the reading has stabilized. The electrode is then placed into the sample solution to be tested to determine its pH level. FA content in the samples was determined by the same method as 2.2. Three parallel samples in each group.

### 2.5 Scanning electron microscope analysis

The bacterial solution in the early stage of the platform was centrifuged, washed three times with PBS, then resuspended and fixed overnight with 2.5% glutaraldehyde phosphate buffer. Then, the samples were fixed with 1% osmic acid solution for 1–2 h. After washing, the bacteria cells were dehydrated using a graded ethanol series, and then dried in a critical point dryer (Quorum k850, Quorum UK Ltd., Nottingham, UK). The samples were fixed on the sample stage using conductive carbon adhesive, gold sprayed for about 30 s using an ion sputtering instrument (Hitachi MC1000, Hitachi Ltd., Tokyo, Japan), and observed under a scanning electron microscope (Hitachi SU3050) to collect images (Ali et al., 2022).

### 2.6 Hemolysis test and growth curve determination

The activated B1-28, *ΔFolA*, *Staphylococcus aureus* and *Bacillus cereus* were passed to the third generation and then the bacterial solutions were diluted to 10^8^ CFU/mL with sterile PBS. The inoculation ring was used to dip a small amount of bacterial solution on the blood agar plate containing 5% defibrinated sheep blood (Solarbio, China) and incubated at 37°C for 48 h. The obvious transparent circle around the pathogenic indicator *Staphylococcus aureus* and *Bacillus cereus* showed a positive hemolysis, which was used as a positive control for the lactic acid bacteria hemolysis test. B1-28 and *ΔFolA* were inoculated at 2% to the MRS broth medium, then the strains were cultured at 37°C with shaking at 180 rpm. The optical density (OD) at 600 nm was measured using a UV–visible spectrophotometer (TU-1810, Beijing, China) at intervals of 2 h, and recorded continuously for 48 h.

### 2.7 Acid and bile salt tolerance test

The viability of B1-28 and *ΔFolA* in low pH and 0.3% bile salt environments was determined by measuring the number of viable colonies (Ali et al., 2023; Jiang et al., 2023). The concentration of cultured bacterial solution was diluted to 10^8^ CFU/mL. The bacterial solutions were centrifuged, then the resulting precipitate were resuspended in sterilized MRS broth medium with pH of 1.5, 2.0, 3.0, 6.25 (original medium pH, control) and 0.3% bile salt. The strains were incubated at 37°C with shaking at 180 rpm. Samples were taken at 0, 1, and 3 h, respectively, and the number of viable bacteria was recorded. The viable bacterial count of the bacterial solution in MRS broth medium at pH 6.25 was used as a control to calculate the survival rate of the bacterial solution at different pH values with the following formula:

$$\mathrm{Survival}\;\mathrm{rate}\;\left(\%\right)=\frac{N1}{N0}\;\times 100\%$$

N0: number of viable bacterial count in 0 h.

N1: number of viable bacterial count in 1 or 3 h.

### 2.8 Adhesion to human colon cancer (Caco-2) cells

The adhesion ability of B1-28 and *ΔFolA* was assessed using the Caco-2 cell line (Li et al., 2023; Liu et al., 2023). Caco-2 cells were inoculated into 6-well plates at a density of 1 × 10^5^ cells per well and cultured to monolayer in complete medium containing Dulbecco’s modified eagle medium (DMEM, Gibco, 11965092), fetal bovine serum (Gibco, 10099141C), penicillin–streptomycin (Gibco, 15140122) and MEM non-essential amino acids solution (Gibco, 11140050). At 37°C and 5% CO_2_, 1 mL of bacterial solution (10^8^ CFU/mL) per well was added to a six-well plate full of Caco-2 cells, then 1 mL DMEM was added into each well. After incubation at 37°C and 5% CO_2_ for 2 h, the non-adherent bacteria were removed by washing with PBS 5 times, adding 0.5 mL 0.25% Trypsin–EDTA (Gibco, 25200072) for digestion for 3 min, adding 1 mL DMEM for repeated blowing, and recording the number of viable bacteria by plate counting method. The adhesion rate of B1-18 and *ΔFolA* to Caco-2 cells was determined by dividing the number of viable adherent bacteria (N2 h) by the initial number of Caco-2 cells or initial number of bacteria (N0 h).

### 2.9 Antibiotic sensitivity test

The susceptibility of B1-28 and *ΔFolA* to 15 antibiotics, including penicillin, ampicillin, and tetracycline, etc., were performed according to the literature-adapted test methods (Wenzler et al., 2023). Briefly, the B1-28 and the *ΔFolA* were passed to the third generation, then the bacterial solutions were diluted to 10^8^ CFU/mL. 100 μL of the diluted bacterial solutions were taken and evenly spread on the MRS solid medium with a coating rod. After letting them stand for 20 min, pieces of drug-sensitive paper were placed equidistantly on the MRS solid medium. After anaerobic incubation at 37°C for 18–24 h, the diameter (mm) of inhibition zones was measured using a vernier caliper.

### 2.10 Antibacterial activity test

The antimicrobial capacities of the strains against six pathogenic bacteria were tested by the agar diffusion method (Chavez-Esquivel et al., 2021). The third-generation activated cultures of B1-28 and *ΔFolA* were diluted to 10^8^ CFU/mL, inoculated with 3%, and cultured for 36 h. After centrifugation, the supernatant was collected. The third-generation activated indicator strain were also diluted to 10^8^ CFU/mL, evenly spread on nutrient agar plates and placed in sterile air for 20–30 min. Subsequently, four Oxford cups were placed on each plate, with 200 μL of supernatant added to three Oxford cups, while an equal amount of sterile MRS broth medium was added to the fourth cup as a control. The plates were incubated at 37°C for 20–24 h, and the diameter of the inhibition circle (mm) was measured by vernier calipers.

### 2.11 Untargeted metabolite detection

Strains B1-28 and *ΔFolA* (10^8^ CFU/mL) were inoculated into sterilized MRS broth and cow’s milk at 6%, respectively, and cultured and fermentation at 37°C for 30 h. Metabolites in MRS Medium supernatant and cow’s milk after fermentation were detected. Six parallel samples in each group. The samples were placed in EP tubes and resuspended with prechilled 80% methanol by well vortex. Then the samples were melted on ice and whirled for 30 s. After the sonification for 6 min, they were centrifuged at 5,000 rpm, 4°C for 1 min. The supernatant was freeze-dried and dissolved with 10% methanol. Untargeted metabolic analysis was performed using a Vanquish UHPLC system (ThermoFisher, Germany) coupled with an Orbitrap Q Exactive^™^ HF mass spectrometer (Thermo Fisher, Germany) in Novogene Co., Ltd. (Beijing, China) platform as described elsewhere (Sellick et al., 2011; Yuan et al., 2012). Samples were injected onto a Hypersil Goldcolumn (100 × 2.1 mm, 1.9 μm) using a 12-min linear gradient at a flow rate of 0.2 mL/min. The eluents for the positive and negative polarity modes were eluent A (0.1% FA in Water) and eluent B (Methanol). The solvent gradient was set as follows: 2% B, 1.5 min; 2–85% B, 3 min; 85–100% B, 10 min;100–2% B, 10.1 min; 2% B, 12 min. Q Exactive^™^ HF mass spectrometer was operated in positive/negative polarity mode with spray voltage of 3.5 kV, capillary temperature of 320°C, sheath gas flow rate of 35 psi and aux gas flow rate of 10 L/min, S-lens RF level of 60, Aux gas heater temperature of 350°C. The raw data files generated by UHPLC–MS/MS were processed using the Compound Discoverer 3.3 (CD3.3, ThermoFisher) to perform peak alignment, peak picking, and quantitation for each metabolite.

### 2.12 Transcription sequencing

RNA was extracted from the logarithmic strains using the RNAprep Pure Cell/Total RNA Extraction Kit (Centrifugal Column Type, Catalog No. DP430), and RNA integrity and quantity were accurately detected using the Agilent 2100 Bioanalyzer (Agilent Technologies, CA, USA). RNA sequencing was performed in the Illumina sequencing platform of Novogene Co., Ltd. (Beijing, China). Differential expression analyses for both conditions/groups were performed using the DESeq2 R package (version 1.20.0). GO enrichment analysis of differentially expressed genes was achieved by GOseq R package software. Statistical enrichment of differentially expressed genes in the KEGG pathway was analyzed using KOBAS software (Mao et al., 2005; Kanehisa et al., 2007).

### 2.13 Statistical analyses

Unless otherwise stated, the data from this study are expressed as mean ± standard deviation (SD). Two-tailed unpaired t-tests were used for two groups and one-way analysis of variance (ANOVA) was used for multiple groups. Excel 2010 software was used to organize basic data, and GraphPad Prism 8.0.2 software was used for data analysis and graphing. Calculated values of *p* < 0.05 were considered statistically significant.

## 3 Results

### 3.1 Screening of human breast milk-derived high FA-producing strains

**Figure 1:**
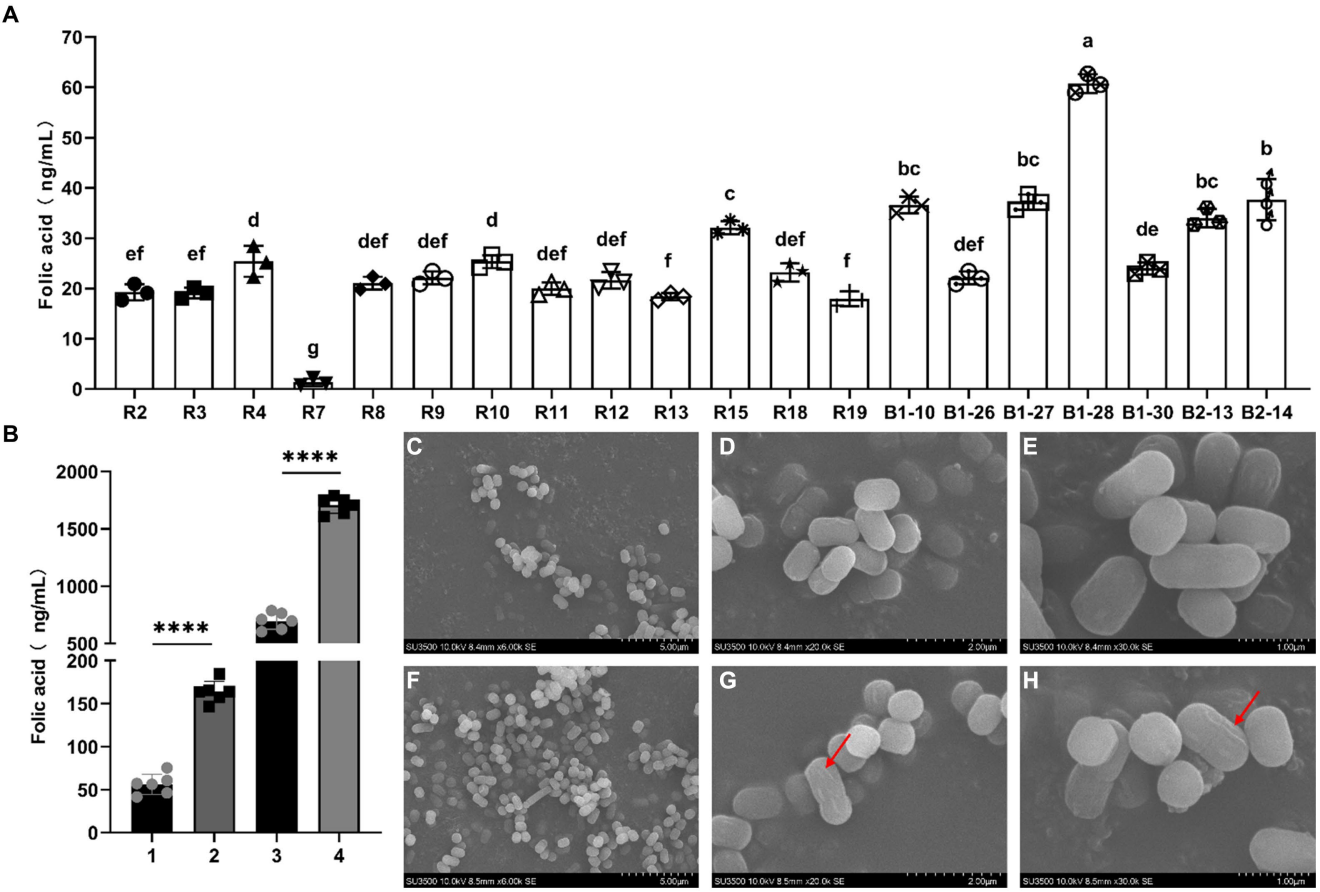
Screening, morphology and FA production abilities of human breast milk-derived strains. **(A)** Screening of FA producing human breast milk-derived strains. The abscissa indicates the strain number, and the ordinate indicates the FA content. **(B)** FA production of the wild-type strain and knockout strain. The abscissa number 1 means FA production of B1-28 in FACM; number 2 means FA production of *ΔFolA* in FACM; number 3 means FA production of B1-28 in milk; number 4 means FA production of *ΔFolA* in milk. **(C–E)** SEM images of B1-28, **(C)** 5.00 μm; **(D)** 2.00 μm; **(E)** 1.00 μm. **(F–H)** SEM images of *ΔFolA*, **(F)** 5.00 μm; **(G)** 2.00 μm; **(H)** 1.00 μm. The results of T-test were expressed as mean ± standard deviation, ****p < 0.0001.

Bromocresol violet was added into FACM to observe the FA production of the tested strains. Among 42 strains of human breast milk-derived strains, the medium solution of 20 strains turned significantly yellow, indicating that the strain had the capacity of producing FA. The 20 strains of human breast milk-derived strains obtained from the initial screening were tested by liquid chromatography mass spectrometry (LC–MS). The results were shown in Figure 1A, all strains produced folic acid with varying yields, and the strain with relatively high FA content was *L. reuteri* B1-28 (60.72 ng/mL), which could be used for further experiments.

### 3.2 FA production and morphological changes in *Limosilactobacillus reuteri* B1-28 and *ΔFolA*

The human breast milk-derived strains B1-28 and *ΔFolA* were cultured in FACM and in cow’s milk to test the FA content. As shown in Figure 1B, the FA production of knockout strain *ΔFolA* was approximately 150 ng/mL in FACM, which was about three times higher than that of the wild-type strain. After fermentation in milk, the folate content of the knockout strain was as high as 1,700 ng/mL, which was about 2.5 folds that of the wild-type strain. This indicates a significant improvement in FA production after *folA* gene knockout of B1-28 strain. Scanning electron microscopy images revealed that both the B1-28 and *ΔFolA* strains exhibited rod-shaped morphology (Figures 1C–H). However, the surface of the *ΔFolA* (Figures 1F–H) was rougher than that of the B1-28 (Figures 1C–E), suggesting that knockout of the *folA* gene affected the surface morphology of the strains.

### 3.3 Evaluation of biological characteristics of B1-28 and *ΔFolA* strains

**Figure 2:**
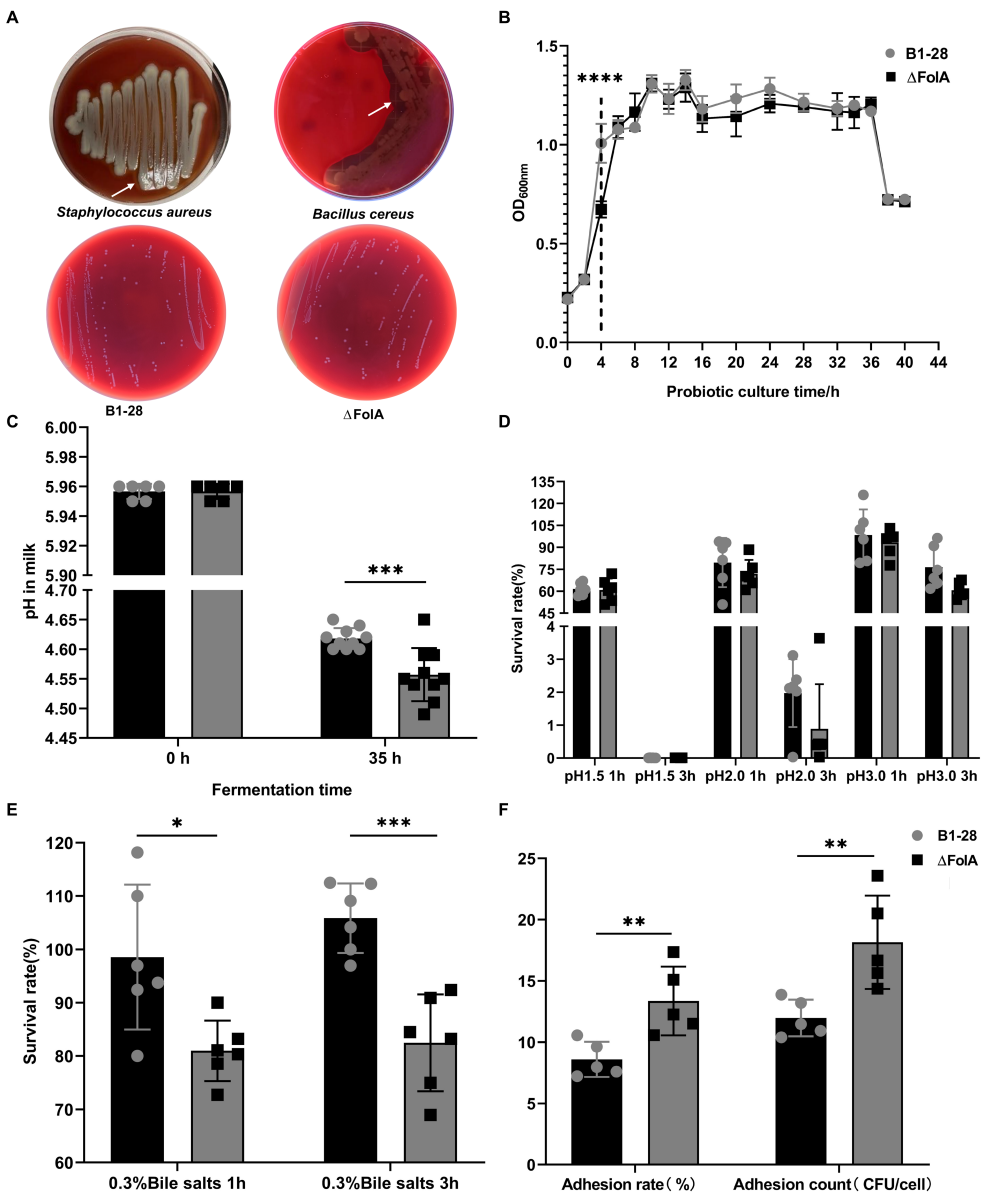
*In vitro* probiotic properties of strains B1-28 and *ΔFolA*. **(A)** Hemolytic activity. **(B)** Growth curve of the strains from 0 to 40 h. **(C)** pH values before and after probiotic fermentation. **(D)** Acid tolerance of strains. **(E)** Bile salt tolerance of strains. **(F)** Adhesion properties of strains. Values are expressed as mean ± standard deviation. * represents statistically significant differences, *p < 0.05, **p < 0.01, ***p < 0.001, ****p < 0.0001. The gray dot represents strain B1-28, and the black square dot represents strain *ΔFolA*.

In Figure 2A, both *Staphylococcus aureus* and *Bacillus cereus* showed obvious hemolytic circles, whereas none of the human breast milk-derived strains exhibited hemolytic activity. The OD600 nm values of B1-28 and *ΔFolA* during 40 h of continuous incubation in MRS broth medium are shown in Figure 2B. The B1-28 grew faster from 2 to 6 h than the *ΔFolA* (*p* < 0.05). The pH values of the broth before and after fermentation were measured as shown in Figure 2C. The pH value of the milk fermented by the *ΔFolA* was significantly lower than that of the milk fermented by the B1-28 (*p* < 0.05). Based on the above experimental results, both strains were tested for acid and bile salt tolerance, and the number of viable bacteria was recorded, as shown in Figure 2D. No difference in the number of viable bacteria were observed between the two strains. Figure 2E shows that the survival rates of the B1-28 and the *ΔFolA* in MRS broth medium containing 0.3% bile salt were higher than 70%, while the B1-28 had a better ability to tolerate bile salts than the *ΔFolA*. Based on the previous scanning electron microscopy results, *ΔFolA* had a rougher bacterial surface than B1-28. *ΔFolA*’s adhesion rate and the number of adherent cells were approximately 1.4 folds higher than those of B1-28 (*p* < 0.01), indicating that *ΔFolA* had a stronger adhesion ability to the human colon cancer cells Caco-2 than B1-28 (Figure 2F).

### 3.4 Antibiotic sensitivity test

Antibiotic sensitivity of probiotics is one of the most important indicators of their safety for use in food (Pierce et al., 2023; Wenzler et al., 2023). In this study, the sensitivities of B1-28 and *ΔFolA* strains to 15 common antibiotics were tested, and the results are presented in Table 1. There was no statistically significant difference between B1-28 and *ΔFolA* in sensitivity to tetracycline and amoxicillin. The diameters of the drug-sensitive circles of B1-28 were significantly smaller than those of *ΔFolA* in penicillin, cefazolin, erythromycin, chloramphenicol, and roxithromycin (*p* < 0.05). Conversely, the diameters of drug-sensitive circles of B1-28 were significantly larger than those of *ΔFolA* in ampicillin, ceftriaxone, augmentin, and enrofloxacin (*p* < 0.05). The *folA* gene knockout strain showed resistance to oxacillin, gentamicin, and neomycin, which was significantly different from B1-28 (*p* < 0.05). Both the B1-28 and the *ΔFolA* showed resistance to kanamycin, streptomycin, norfloxacin, enoxacin and polymyxin B, suggesting that up-regulation of *folA* expression as a target of drug action affects microbial susceptibility to antibiotics (Shi et al., 2021; Angermayr et al., 2022).

**Table 1:** Antibiotic sensitivity test results.

Name of antibiotic	Content/tablet	Evaluation criteria (mm)		B1-28			*ΔFolA*	
		S	I	R	Zone of inhibition (diameter, mm)	Sensitivity	Zone of inhibition (diameter, mm)	Sensitivity
Penicillin	10 U	≥15.00		≤14.00	22.19 ± 0.36^a^	S	30.69 ± 0.21^b^	S
Oxacillin	1 μg	≥18.00	11.00–12.00	≤10.00	11.65 ± 0.57^a^	I	0.00^b^	R
Ampicillin	10 μg	≥17.00	14.00–16.00	≤13.00	28.87 ± 0.34^a^	S	27.53 ± 0.43^b^	S
Cefazolin	30 μg	≥18.00	15.00–17.00	≤14.00	27.10 ± 0.28^a^	S	30.98 ± 0.61^b^	S
Ceftriaxone	30 μg	≥21.00	14.00–20.00	≤13.00	32.44 ± 0.53^a^	S	28.94 ± 0.19^b^	S
Gentamicin	10 μg	≥15.00	13.00–14.00	≤12.00	13.17 ± 0.21^a^	I	0.00^b^	R
Kanamycin	30 μg	≥18.00	14.00–17.00	≤13.00	0.00^a^	R	0.00^a^	R
Streptomycin	10 μg	≥15.00	12.00–14.00	≤11.00	0.00^a^	R	0.00^a^	R
Neomycin	30 μg	≥17.00	13.00–16.00	≤12.00	14.75 ± 0.55^a^	I	0.00^a^	R
Tetracycline	30 μg	≥19.00	15.00–18.00	≤14.00	21.87 ± 0.01^a^	S	21.95 ± 0.41^a^	S
Erythromycin	15 μg	≥23.00	14.00–22.00	≤13.00	18.87 ± 0.44^a^	I	20.10 ± 0.34^b^	I
Norfloxacin	10 μg	≥17.00	13.00–16.00	≤12.00	0.00^a^	R	0.00^a^	R
Enoxacin	10 μg	≥18.00	15.00–17.00	≤14.00	0.00^a^	R	0.00^a^	R
Amoxicillin	20 μg	≥18.00	14.00–17.00	≤13.00	25.67 ± 0.33^a^	S	25.25 ± 0.41^a^	S
Chloramphenicol	30 μg	≥18.00	13.00–17.00	≤12.00	21.41 ± 0.61^a^	S	24.67 ± 0.44^b^	S

S (Sensitive): the recommended dose achieves good inhibition; I (Intermediary): the antimicrobial strain is close to levels achievable in blood and tissues, but the therapeutic efficacy may be lower than that of sensitive strains; R (Resistant): there is no significant inhibition at normal doses. The same letter in the same row of markers indicates a non-significant difference (*p* > 0.05), and different letters indicate a significant difference (*p* < 0.05).

### 3.5 Antibacterial activities of culture supernatant

The fermentation broths of strains B1-28 and *ΔFolA* were used to evaluate their antibacterial activities (Table 2). Both B1-28 and *ΔFolA* exhibited specific inhibitory effects on different pathogenic bacteria, with varied degrees of antibacterial activities. Specifically, B1-28 was significantly better than *ΔFolA* in inhibiting *Staphylococcus aureus* and *Salmonella typhimurium* (*p* < 0.05). Conversely, *ΔFolA* exhibited stronger inhibitory capabilities than B1-28 against *Escherichia coli*, *Salmonella enteritidis*, and *Bacillus cereus* (*p* < 0.05).

**Table 2:** Antibacterial activity test results of strains.

Strain number	*Escherichia coli*	*Staphylococcus aureus*	*Salmonella enteritidis*	*Salmonella typhimurium*	*Listeria monocytogenes*	*Bacillus cereus*
	Inhibitory ring diameter (mm)					
B1-28	19.21 ± 0.68^a^	18.57 ± 0.50^a^	18.23 ± 0.32^a^	18.17 ± 0.41^a^	22.60 ± 2.82^a^	20.74 ± 1.21^a^
*ΔFolA*	22.37 ± 1.35^b^	16.40 ± 0.09^b^	20.50 ± 0.85^b^	16.80 ± 0.20^b^	22.71 ± 1.36^a^	26.03 ± 0.41^b^

Values are expressed as mean ± standard deviation. T-test. The same column labeled with the same letter indicating a non-significant difference (*p* > 0.05), and different letters indicating a significant difference (*p* < 0.05).

### 3.6 Analysis of differential metabolites in fermentation samples

**Figure 3:**
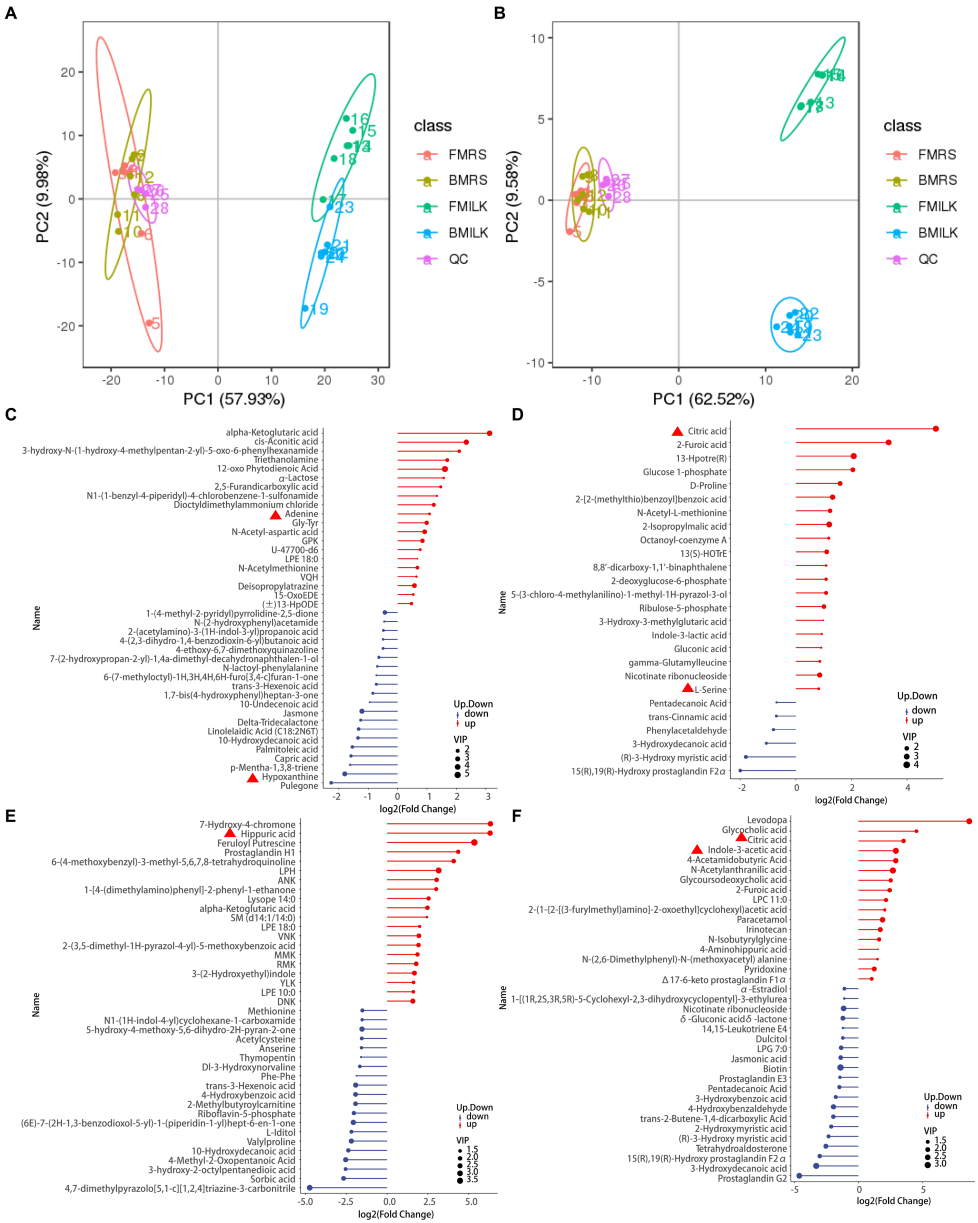
Screening results of differential metabolite in fermentation samples. **(A)** PCA plot in positive ion mode; **(B)** PCA plot in negative ion mode; scatters with different colors indicate samples from different experimental subgroups; FMRS: *ΔFolA* fermentation medium; BMRS: B1-28 fermentation medium; FMILK: *ΔFolA* fermented milk; BMILK: B1-28 fermented milk. **(C)** Matchstick diagram of differential metabolites in MRS broth fermentation broth in positive ion mode. **(D)** Matchstick diagram of differential metabolites in MRS broth fermentation broth in negative ion mode. **(E)** Matchstick diagram of differential metabolites in fermented milk in positive ion mode. **(F)** Matchstick diagram of differential metabolites in fermented milk in negative ion mode. The color of the dots represents up-regulation or down-regulation, blue represents the downregulation, and red represents up-regulation; the length of the rods represents the magnitude of the log2(Fold Change); and the size of the dots represents the magnitude of the VIP value.

The PCA plots of the B1-28 and *ΔFolA* in positive and negative ion modes during the growth period in MRS broth medium and cow’s milk are displayed in Figures 3A,B. They both showed obvious regular differences. A total of 619 positive ion mode metabolites and 251 negative ion mode metabolites were identified in the 24 samples (Supplementary Table S1). Differential metabolites were screened by setting threshold values as VIP > 1.0, 1.2 or FC < 0.833 and *p*-value <0.833 (Wen et al., 2017). The values of the screened differential metabolites are shown in Table 3 and the top 20 differential metabolites after up- and down-regulation are shown in Figures 3C–F. The B1-28 and *ΔFolA* produced metabolic differential products dominated by organic acids in MRS broth in positive and negative ion modes, including citric acid, L-serine, adenine, α-ketoglutaric acid and other differential metabolites. And, in milk, the B1-28 and *ΔFolA* produced 60 differential metabolites, such as citric acid and indole-3-acetic acid, in negative ion mode, and 143 significantly up-regulated differential metabolites, such as hippuric acid, in positive ion mode.

**Table 3:** Results of screening for metabolite differences.

Compared samples	Num. of total ident.	Num. of total sig.	Num. of sig. up	Num. of sig. down
FMRS vs. BMRS_pos	45.04	46	21	25
FMILK vs. BMILK_pos	45.04	143	43	100
FMRS vs. BMRS_neg	45.04	43	37	6
FMILK vs. BMILK_neg	45.04	60	17	43

Compared samples: pairs of samples compared, the former to the latter; Num of Total Ident: total metabolite identification; Num of Total Sig: total number of metabolites with significant differences; Num of Sig Up: total number of metabolites significantly up-regulated; Num of Sig down: total number of metabolites significantly down-regulated.

**Figure 4:**
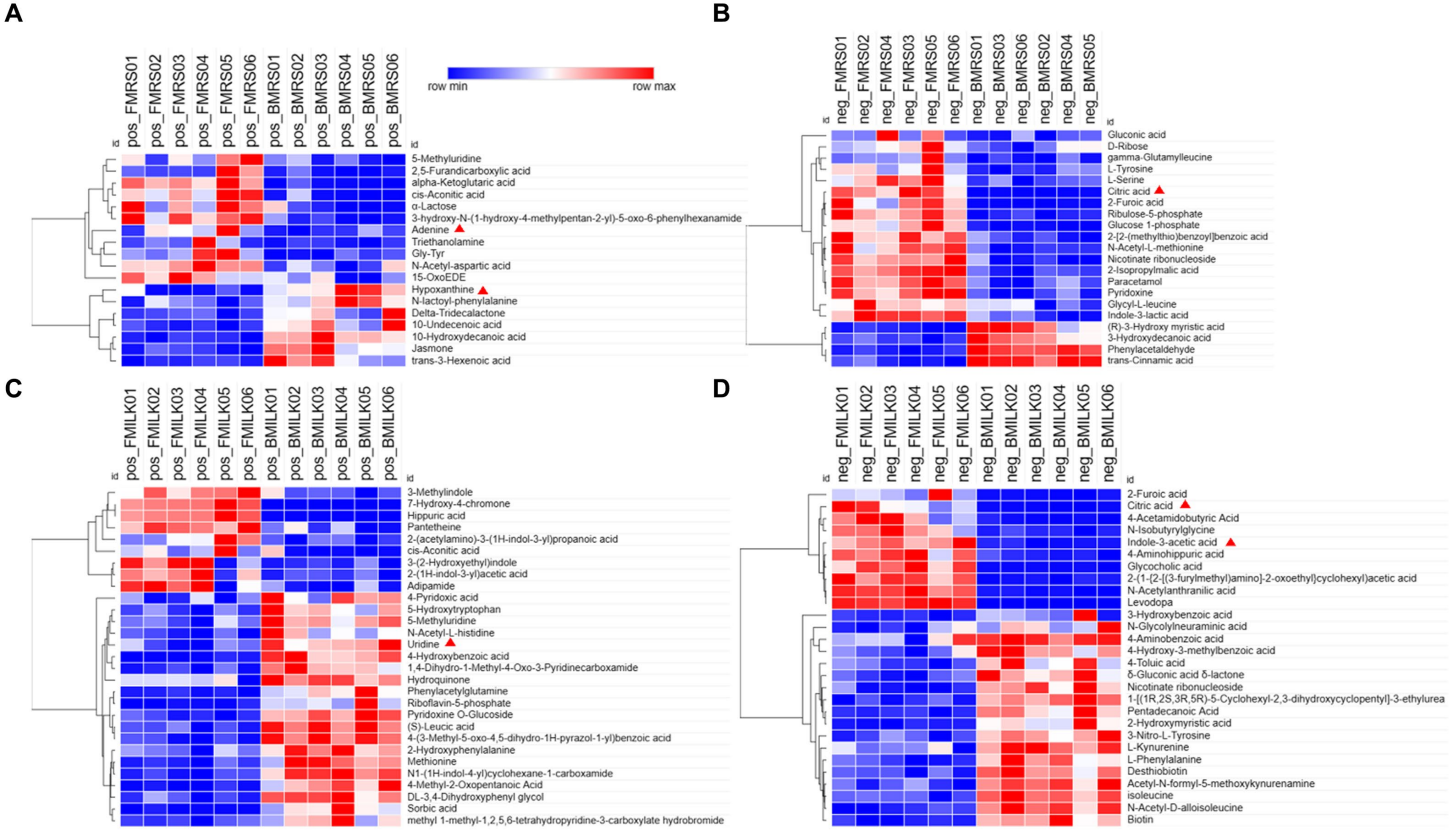
Heat maps of differential metabolites in fermentation samples. **(A,B)** Heat maps of differential metabolites in MRS fermentation broth in positive and negative ion modes. **(C,D)** Heat maps of differential metabolites in fermented milk in positive and negative ion modes. Clustering is done vertically for samples and horizontally for metabolites, with shorter cluster branches representing higher similarity. Horizontal comparisons show the relationship between the clustering of metabolite contents between groups. FMRS: *ΔFolA* fermentation medium; BMRS: B1-28 fermentation medium; FMILK: *ΔFolA* fermented milk; BMILK: B1-28 fermented milk. Sample numbers 01–06 represent six parallel samples in this group.

Metabolomics analysis is an effective method to compare the differential metabolite expression between wild-type and knockout strains. Hierarchical clustering analysis of the two sets of differential metabolites obtained showed that strains B1-28 and *ΔFolA* fermented different media into different differential metabolites, including organic acids and their derivatives, organic oxids, indoles and their derivatives, carboxylic acids and their derivatives of purines. These differences in metabolite abundance are shown in the clustered heatmap (Figures 4A–D). It can be seen from the heat map that the metabolic patterns were similar within the same group. After fermentation, the *ΔFolA* produced higher levels of citric acid, pyridoxine, 2-furanic acid, L-serine, and L-tyrosine than B1-28 (*p* < 0.01). Moreover, the strains produced more metabolites by fermentation in cow’s milk than those in the basic medium, suggesting that some metabolites are more readily activated in cow’s milk. Fermented milk is considered to be the most ideal vehicle for delivering probiotics into the body, which can perfectly combine the health benefits of probiotics and milk (Sun et al., 2023). By comparing the different metabolites, no toxic metabolites were found (Supplementary Table S1), which need to be evaluated in further safety experiments.

### 3.7 Metabolic pathway analysis of differential metabolites in fermented broth/milk

**Figure 5:**
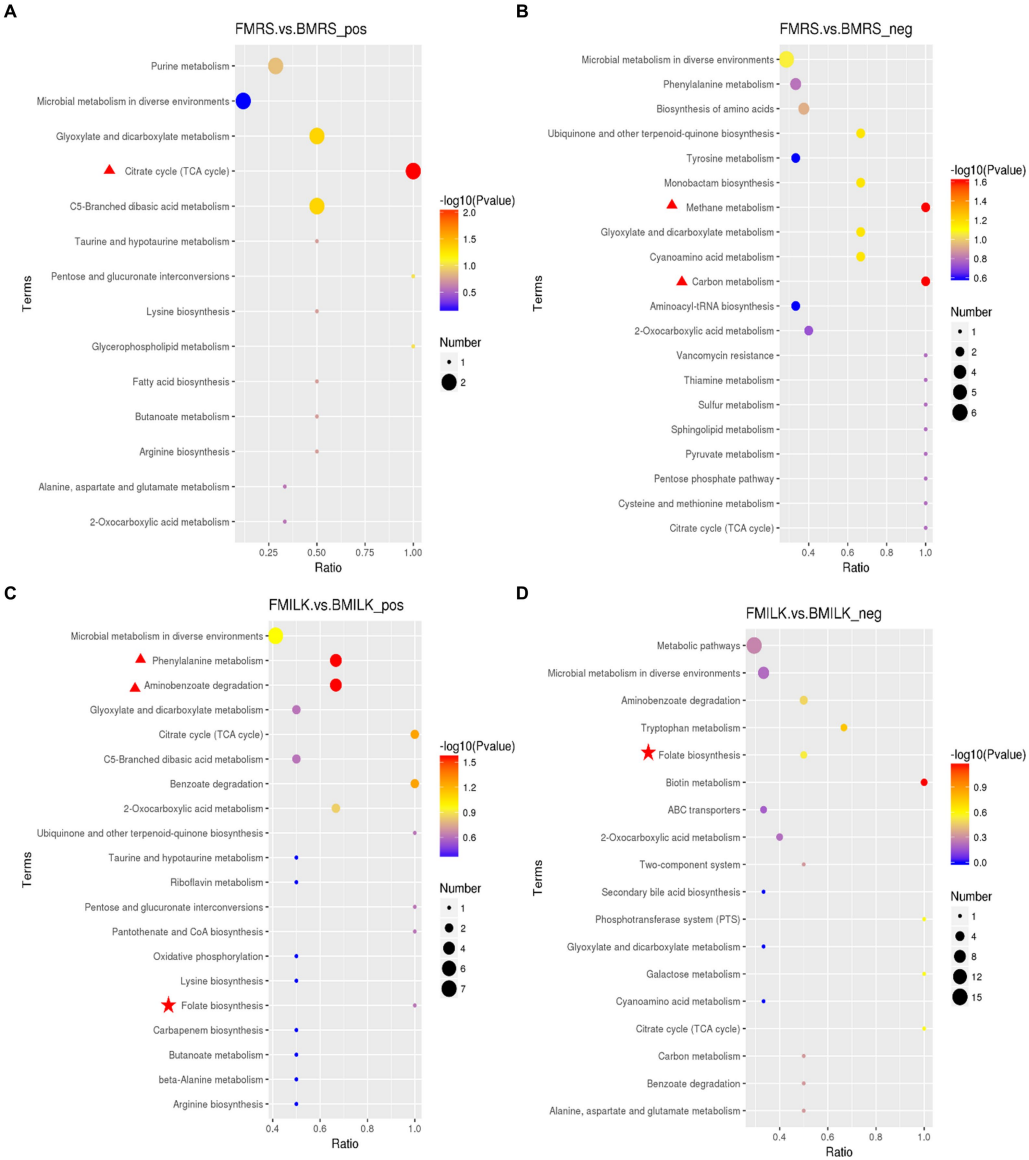
KEGG pathway analysis. **(A)** KEGG pathway of MRS fermentation broth in the positive ionic mode. **(B)** KEGG pathway of MRS fermentation broth in the negative ionic mode. **(C)** KEGG pathway of fermented milk in the positive ionic mode. **(D)** KEGG pathway of fermented milk in the negative ionic mode. The horizontal coordinate in the graph is x/y (the number of differential metabolites in the corresponding metabolic pathway/ the number of total metabolites identified in the pathway), larger values indicate higher enrichment of differential metabolites in the pathway. The colors of the dots represent the *p*-value of the hypergeometric test, smaller values indicate greater reliability and statistical significance of the test. FMRS: *ΔFolA* fermentation medium; BMRS: B1-28 fermentation medium; FMILK: *ΔFolA* fermented milk; BMILK: B1-28 fermented milk.

KEGG is a powerful tool for metabolic analysis and metabolic network studies in organisms. Pathway enrichment enabled the identification of the most prominent biochemical metabolic pathways in which the differential metabolites are involved, as shown in Figures 5A–D, including the citrate cycle, biosynthesis of amino acids, FA biosynthesis, purine metabolism, and butyric acid metabolism, and other metabolic pathways. In MRS broth, significant differences were observed in the citrate cycle (*p* = 0.008), glyoxylate and dicarboxylate metabolism (*p* = 0.045), and C5-branched dicarboxylic acid metabolism (*p* = 0.045) in positive ion mode, while in the negative ion mode, differences observed in methane and carbon metabolism (*p* = 0.025) were related to citric acid, L-serine and L-tyrosine, which was consistent with the results of the above differential metabolites. In cow’s milk, significant differences were observed in phenylalanine metabolism (*p* = 0.028) and aminobenzoic acid degradation (*p* = 0.028), and the enriched metabolites included phenylglyoxylic acid, phenylacetylglutamine, hippuric acid, p-hydroxybenzaldehyde, hydroquinone, and 4-hydroxybenzoic acid in the positive ionic mode. In negative ionic mode, the metabolites enriched in FA biosynthesis included dihydrofolate and 4-aminobenzoic acid, and the reason for no significant difference is that FA is unstable and prone to oxidative decomposition (Mahara et al., 2019). These metabolic pathways for the *folA* gene to exert probiotic efficacy *in vivo*.

### 3.8 Transcription sequencing analysis

**Figure 6:**
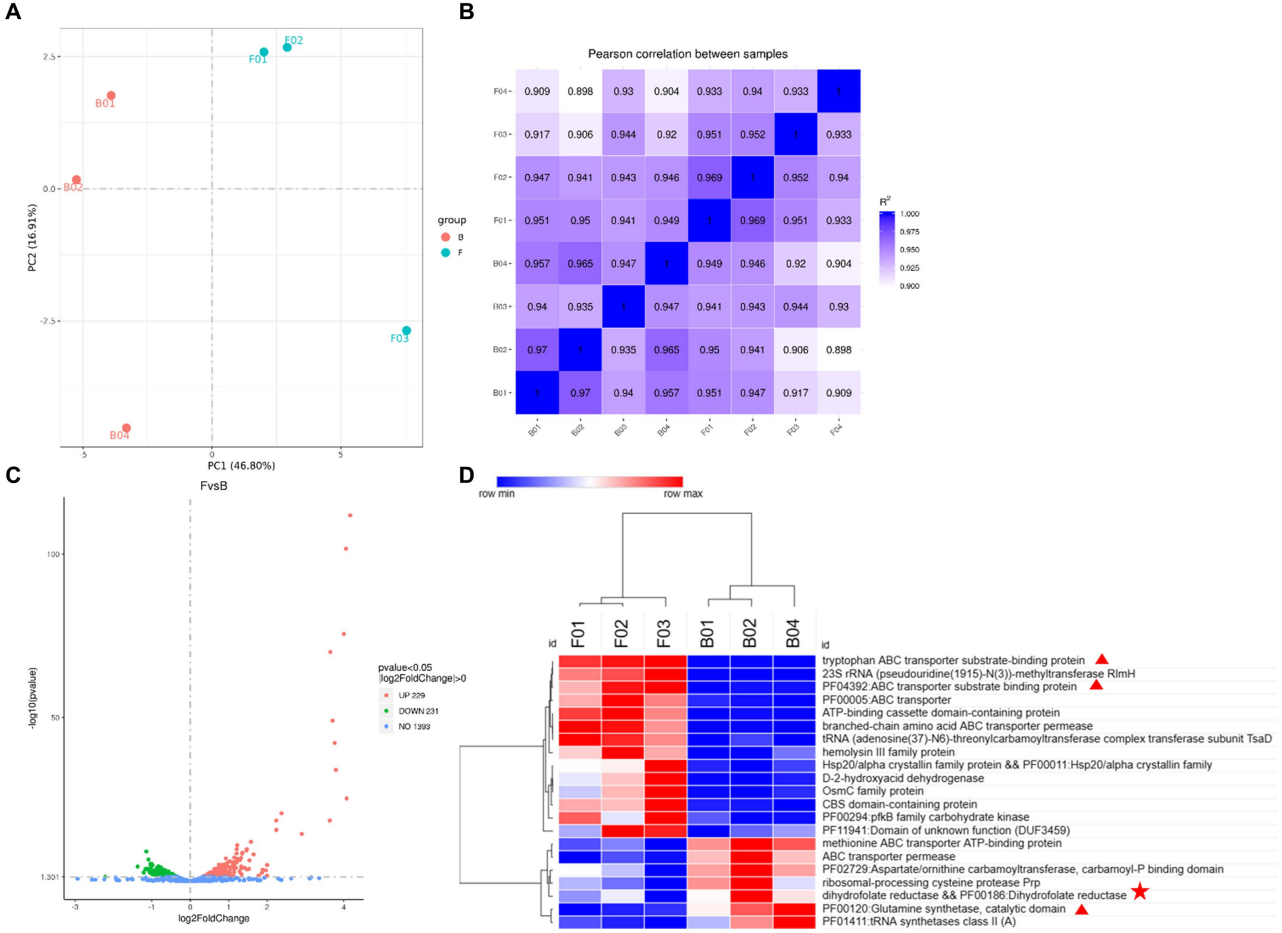
Transcriptional differential gene analysis. **(A)** PCA plot, in which samples between groups are dispersed and samples within groups are clustered together. **(B)** Gene expression correlation map, the closer the correlation coefficient is to 1, the higher the similarity in expression patterns between samples. **(C)** Differential gene volcano map, the horizontal coordinates of the graphs indicate the fold change in gene expression between the treatment and control groups (log2FoldChange), and the vertical coordinates indicate the significance level of the difference of gene expression between the treatment and control groups (−log10 padj or -log10 *p*-value). Up-regulated genes are represented by red dots and down-regulated genes are represented by green dots. **(D)** Differential gene function heatmap. Cluster analysis was conducted on differential gene sets, where genes with similar expression patterns were clustered together. When compared horizontally, red indicates high gene expression, and blue indicates low gene expression.

To investigate the expression of the strains at the RNA level after *folA* knockout, B1-28 and *ΔFolA* were analyzed by transcriptome sequencing. PCA analysis was conducted on the gene expression values (FPKM) of all samples, as shown in Figure 6A. Samples were scattered between groups and clustered within groups. The cDNA libraries were constructed by Illumina sequencing with three or more replicates per strain. The Q20 of each library was >97.84%, and the Q30 was >93.71%, indicating good sequencing results. The correlation coefficients of samples within and between groups were calculated based on the FPKM values of all the genes in each sample, where a higher correlation coefficient between samples (closer to 1) indicates higher similarity in expression patterns between the samples. The B1-28 and *ΔFolA* samples were divided into two distinct categories with good intra-group correlation (Figure 6B), where the Pearson correlation coefficient quadratic sum R2 exceeding 0.898 indicates relatively ideal sampling and experimental conditions for subsequent transcriptome analyses. The differential genes with changes in expression were screened in the sequencing library, and the complete list of differential genes is presented in Supplementary Table S2. The results are represented in the Volcano plot in Figure 6C. The *ΔFolA* showed significant differences in 460 genes compared to the B1-28, of which 229 genes were up-regulated and 231 genes were down-regulated, suggesting that knockout of the *folA* gene influenced the transcriptional level of the entire gene. Cluster analysis of the differential gene set is illustrated in Figure 6D. The major up-regulated genes were associated with the ABC transporter protein family, while the downregulation of the expression of genes encoding dihydrofolate reductase and glutamine synthetase was associated with knockout of the strain’s dihydrofolate reductase-encoding gene, *folA*.

**Figure 7:**
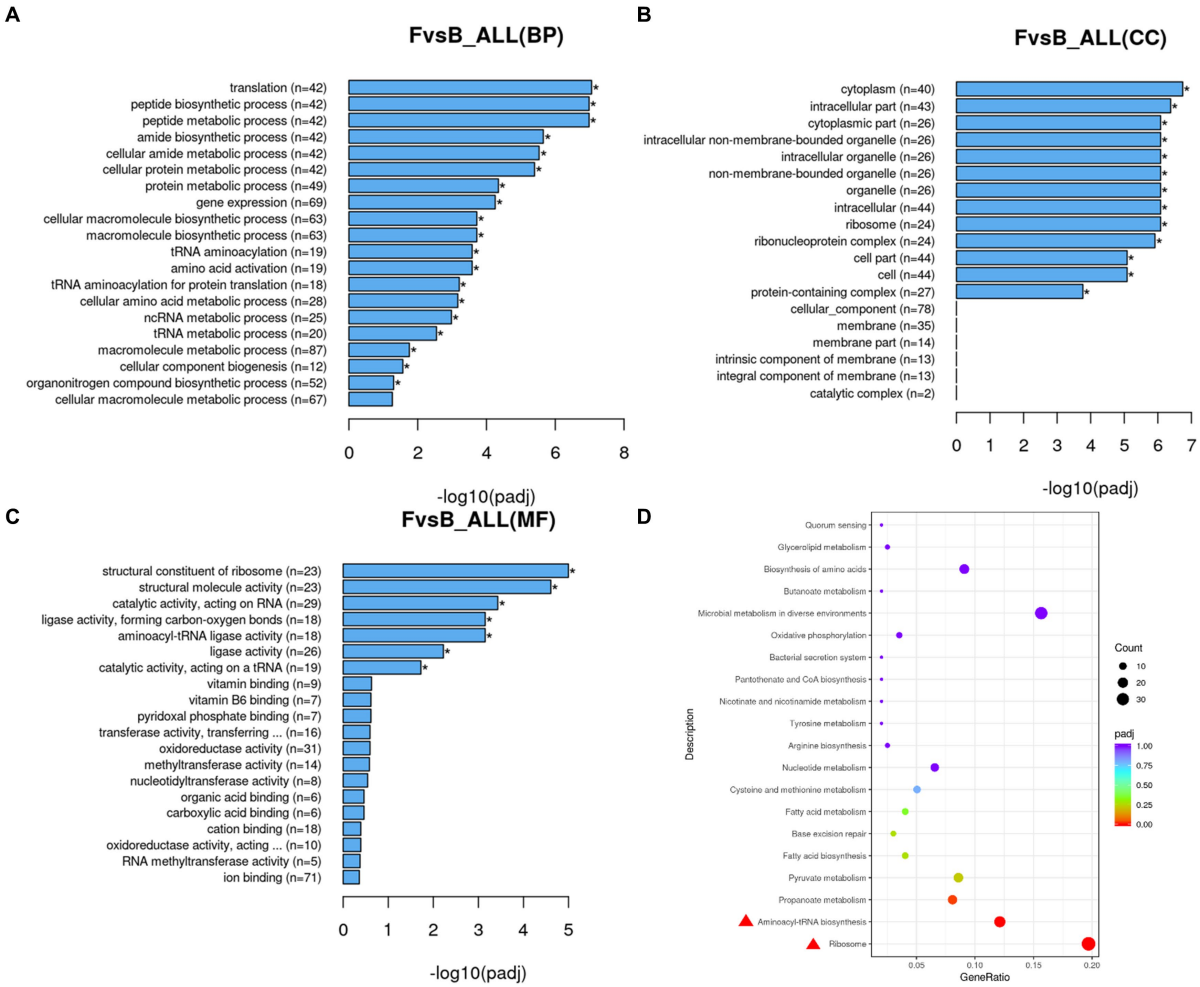
Enrichment analysis diagram. **(A)** GO enrichment histogram of DEGs in biological process. **(B)** GO enrichment histogram of DEGs in cellular components. **(C)** GO enrichment histogram of differential genes in molecular function. n: the number of differential genes annotated to the GO number and the functional analysis of transcriptional DEGs. **(D)** KEGG pathway was plotted as a scatter plot, the horizontal coordinate of the plot is the ratio of the number of differential genes annotated to the KEGG pathway to the total number of differential genes, and the vertical coordinate is the KEGG pathway. The scatter plot is shown with different colors and sizes of the dots, with the colors showing a gradual change from purple to red, the redder the color, the more significant the enrichment is; the bigger the dot, the more genes are enriched.

The Gene Ontology (GO) function was analyzed on 460 differentially expressed genes, as shown in Figures 7A–C. In B1-28 and *ΔFolA*, differentially expressed genes (DEGs) was mainly involved in RNA processing, ribosomal structural components, organic acid transport and aminoacyl-tRNA ligase activity. The results showed that the indirect transcriptional regulation target of folA affected the formation of nucleotides and purines, and the significant increase of citric acid and other organic acid metabolites could improve the strain’s ability to inhibit *Escherichia coli* (Ji et al., 2023). The down-regulated genes may be the result of the target-dependent or compensatory response to the normal function of *folA*, which can not directly prove that *folA* promotes or suppresses the expression of these genes. Therefore, further experimental validation and functional studies are needed to determine the exact role of *folA* in the metabolic process.

To further analyze the impact of *folA* gene knockout on metabolic pathways of strains, 229 up-regulated genes and 231 down-regulated genes were analyzed for metabolic pathways by KEGG (Supplementary Table S3). Up-regulated genes were primarily in pathways such as amino acid biosynthesis, and secondary metabolite biosynthesis, while down-regulated genes were primarily in in the microorganisms in the pathways of nucleotide metabolism, pyrimidine metabolism, purine metabolism, carbon metabolism, and ABC transport protein. On this basis, the differential expression genes annotated by KEGG were further explored (Figure 7D). The enzymes encoded by differential genes were mainly involved in ribosome, aminoacyl-tRNA biosynthesis, and propanoate metabolism, and the expression of EDGs was down-regulated in ribosomal and aminoacyl-tRNA biosynthesis (Padj <0.001). The DEGs enrichment in propionic acid metabolism was significantly higher than that in the control group (*P*adj = 0.027), which was consistent with the functional analysis of GO. These genes and metabolic processes discovered through screening are related to amino acid, ribosomal, and purine synthesis pathways. The results indicate that *folA* gene knockout had a great impact on amino acids metabolic pathways and folate biosynthesis pathways in human breast milk-derived *L. reuteri* B1-28 and had a regulatory effect on genes involved in FA synthesis.

## 4 Discussion

Probiotics, which refer to the intake of active microorganisms in sufficient quantities that are beneficial to host health (Hill et al., 2014), are biological ingredients commonly used in many functional fermented foods (Tripathi and Giri, 2014). Several studies have shown that lactic acid bacteria (LAB) such as industrial fermentation strains like *Lactococcus lactis* and *Lactobacillus lactis* have a stronger capacity to produce active FA (LeBlanc et al., 2020). The human breast milk-derived *L. reuteri* strain used in this study is a potential probiotic candidate with excellent prebiotic properties derived from the human breast milk of healthy mothers, which is closely related to human health. With the development and application of probiotic breeding technology, probiotics can be transformed according to the designer’s own ideas, so as to develop personalized probiotics. Wang et al. (2021) efficiently screened strains with high tolerance to bile salts by knocking out the bsh1 or bsh3 genes. The development of gene editing technology has provided the necessary support for the next generation of probiotics. In order to increase the production of natural FA and meet the human demand, increasing the FA production in probiotics through molecular breeding is the focus of current research (King et al., 2015; Chen et al., 2019). Hill et al. (2023) subcloned *folA*, the FA gene of strain Humboldt, into the TransBac vector and conducted experiments such as growth phenotypic evaluation on the mutant containing Humboldt *folA* subclone and the pFE604 clone with the *folA* gene knocked out, which confirmed the *in vivo* function of *folA* gene from Humboldt strain in producing FA biosynthesis products. Sybesma et al. (2003) showed that overexpression of the *folKE* gene in *Lactobacillus lactis* led to overexpression of DHPPPK and GCHI encoded by the *folKE* gene, and a threefold increase in total FA production. In this study, through molecular breeding, the FA production capacity of *ΔFolA* was 2–3 folds that of the original strain B1-28.

Metabolomics and transcriptomics are emerging omics technologies after genomics and proteomics, and they are important technologies that can reveal the phenomena and processes of life activities through qualitative and quantitative analyses of metabolites of living organisms (Pan et al., 2021). In this study, the dynamic changes of metabolites in B1-28 and *ΔFolA* strains after fermentation in MRS broth and cow’s milk were determined using untargeted metabolomics technique. Transcriptomics can be used to study gene transcription in strains and the law of transcription regulation, to predict protein expression level and function, and to provide an important reference for the identification and quantification of proteins, in which mRNA is the intermediary of gene expression, and protein is the executor of gene function (Echegaray et al., 2023). In our transcriptomic analysis, differential genes were significantly enriched in the ABC transport protein pathway. ABC transport proteins are a large and ancient family of transmembrane transport protein that play important physiological roles by using the energy generated by ATP hydrolysis to transport various substances bound to them across membranes, such as alkanes, amino acids, antibiotics, and other substances (Qu et al., 2020). In this study, we enriched the gene for the substrate binding protein of tryptophan ABC transporter protein. With the continuous progress of biological macromolecule structure analysis technology, the crystal structures of several ABC transport proteins have been successfully analyzed, including Sav1866 multidrug outward transport protein from *Staphylococcus aureus*, ModBCA MoO4/WO4 inward transport protein from Archaeoglobus fulgidus, and BtuCD vitamin B12 inward transport protein from *Escherichia coli* (Hollenstein et al., 2007). The enrichment of ABC transporter substrate-binding protein genes was positively correlated with the metabolites detected in metabolism, such as indole derivatives and kynurenic acid (Supplementary Table S4). Tryptophan metabolites act as intercellular signals in the regulation of intestinal inflammation, mucosal immunity, and barrier function (Zhang et al., 2020b). Up-regulated indole-3-lactic acid in metabolites regulates intestinal immunity and inhibits the occurrence of intestinal cancer (Han et al., 2023). The up-regulated expression of genes encoding ribonucleoprotein complex and ribosome structural components is consistent with the results of increased nucleotide metabolism and ribosomal expression detected in the metabolome. Aminoacyl tRNA is a kind of tRNA that binds to its corresponding amino acids. Its duty is to transfer amino acids to the ribosome and add specific amino acids to the corresponding tRNA (Rubio Gomez and Ibba, 2020). In this study, the down-regulated expression of aminoacyl-tRNA biosynthesis pathway is consistent with the down-regulation of ribosome expression. FA is involved in ribosome and other metabolic pathways, indirectly indicating the key role of *folA* gene in aminoacyl-tRNA biosynthesis pathway. Phosphofructokinase B (PfkB) belongs to the ribokinases family that uses phosphorylated sugars as substrates and catalyzes the conversion of fructose-6-phosphate to fructose-1,6-bisphosphate (Gao et al., 2021). Expression of the pfkB family carbohydrate kinase genes was upregulated in *ΔFolA*, suggesting possible involvement in glycolysis and the pentose phosphate pathway, as well as in supporting processes such as cell growth and differentiation, and the immune response.

The metabolic change process of fermentation broth is jointly regulated by a variety of substances and reactions. Overall judgment cannot be made only based on the levels of one single substance. By comparing with the KEGG database, genes were classified according to their participation in pathways or functions, revealing the metabolic pathways involved in metabolite metabolism (Xie et al., 2020; Wang et al., 2022). Thus, the impact of the biological metabolic process of the fermentation broth of the B1-28 and *ΔFolA* can be evaluated from a macroscopic point of view. In recent years, researchers around the world have carried out extensive research on the relationship between organic acids and the human body. Organic acids have been found with various effects such as anti-virus, anti-fatigue, softening blood vessels, accelerating metabolism, stimulating the secretion of digestive juices, promoting gastrointestinal peristalsis, facilitating the absorption of calcium and iron ions, reducing the generation of peroxides in brain tissue, and maintaining the acid–base balance in human body (Hagenbeek et al., 2020; Tran and Zhao, 2022). Major organic acids include citric acid, malic acid, and tartaric acid, etc. Citric acid, as an important compound in the tricarboxylic acid cycle, supplies energy for biological activities through the tricarboxylic acid cycle (Yoshida and Yokoyama, 2012). By conducting a correlation analysis of differential metabolites and pathways, the metabolic pathways related to the citrate cycle, amino acid biosynthesis, FA biosynthesis, purine metabolism, and propionic acid metabolism, were increased in expression in the *ΔFolA* compared to those in B1-28. *ΔFolA* may therefore be more beneficial for human metabolism and other activities, compared to B1-28.

*Limosilactobacillus reuteri* PSC102 showed good acid and bile salt tolerance without producing harmful enzymes, had no haemolytic characteristics, and significantly inhibited the growth of enterotoxin-producing *Escherichia coli* (Ali et al., 2023). In this study, strains B1-28 and *ΔFolA* exhibited no hemolytic characteristics and *ΔFolA* significantly inhibited the growth of *Escherichia coli* (*p* = 0.0015), the antibacterial abilities of strains B1-28 and *ΔFolA* against *Escherichia coli* and *Staphylococcus aureus* were higher than those of lactic acid bacteria isolated from yak feces (Zhang et al., 2022). Characteristics related to gastrointestinal survival and intestinal colonization include resistance to acidic pH, bile salt hydrolysis, etc. Gastric acid secretion constitutes the primary defense mechanism against most ingested microbes, and different types of lactic acid bacteria have varying degrees of acid and bile salt tolerance (Lavilla-Lerma et al., 2013). In this study, after exposure to pH 3.0 for 3 h, the survival rates of strains B1-28 and *ΔFolA* were similar to those of other lactic acid bacteria isolated from yak fecal samples, FY1 (69.96%), FY2 (68.29%), FY3 (68.86%), and FY4 (68.59%). However, after culturing for 3 h under 0.3% bile salt conditions, the survival rate of strain B1-28 was higher than that of FY3, FY2, and FY4 strains (Zhang et al., 2022). These data show that strain B1-28 exhibits excellent tolerance to bile salt environments. Strains with good antibiotic tolerance can be used as additives to antibiotic-containing feed and can survive in organisms containing antibiotics. The *folA* gene is not strictly a drug-resistant gene (Li and Wang, 2021), but is associated with the intrinsic mechanism of drugs. In the present study there were certain differential changes in antibiotic sensitivities between *ΔFolA* and B1-28. The *ΔFolA* showed resistance to oxacillin, gentamicin, and neomycin, compared to the B1-28. This result is consistent with previous reports that lactic acid bacteria typically exhibit high resistance to kanamycin and streptomycin (Pradhan et al., 2020). Therefore, understanding the antibiotic properties of probiotics is an important indicator for their ability to exert the function of their probiotic precursor, and appropriate products can be developed based on the characteristics of probiotics against antibiotics. Adhesion ability is a prerequisite for probiotics to exert their beneficial effects in colonizing the intestines (Han et al., 2021). Studies by Ali et al. (2023) on the adhesion ability of *L. reuteri* PSC102 found that after co-culturing the strain with Caco-2 cells for 3 h, its adhesion rate was 4.03% (± 0.15), which is lower than the adhesion rates of the human breast milk-derived strains in this study. In conclusion, the human breast milk-derived strains B1-28 and *ΔFolA* have good biological characteristics and are making them potential probiotic candidates for developing folate-rich functional foods.

## 5 Conclusion

This study used probiotic experiment and omics analysis to explore the difference between FA-producing human breast milk-derived *L. reuteri* B1-28 and *ΔFolA*. Our results suggest that the folate production of *ΔFolA*, which was constructed by molecular breeding technique, was 2.5 folds that of B1-28. Probiotic experiments showed that B1-28 and *ΔFolA* exhibited no hemolytic activity, while *ΔFolA* had rougher surface and significantly enhanced adhesion ability, and showed significant differences in drug resistance and bacteriostatic characteristics. This study reveals that folA gene affected the differential changes of human breast milk-derived *L. reuteri* B1-28 through metabonomics and transcriptome analysis. The results showed that the expression of *folA*-related genes was down-regulated, and DEGs were mainly involved in ABC transport and amino acid biosynthesis. The differential metabolites of citric acid and α-ketoglutaric acid were the intermediates of tricarboxylic acid cycle, which participated in the synthesis of amino acids, vitamins, organic acids and energy metabolism, and enhanced the inhibition of pathogenic bacteria. In conclusion, this study revealed the differences in physiology, metabolism and transcription of human breast milk-derived probiotics before and after treatment with molecular breeding technology, which is expected to provide new ideas for exploring the FA biosynthesis pathway in human breast milk-derived probiotics by molecular breeding techniques.

## Data availability statement

The original contributions presented in the study are included in the article/Supplementary material, further inquiries can be directed to the corresponding author.

## Author contributions

YuJ: Data curation, Formal analysis, Investigation, Software, Validation, Writing – original draft. XL: Data curation, Formal analysis, Investigation, Supervision, Writing – review & editing. WZ: Investigation, Validation, Writing – original draft. YaJ: Investigation, Methodology, Validation, Writing – original draft. KY: Writing – review & editing. LL: Writing – review & editing. MZ: Writing – review & editing. WQ: Writing – review & editing. JZ: Writing – review & editing. MD: Writing – original draft. XF: Writing – original draft. XD: Writing – original draft. HC: Writing – original draft. TJ: Writing – review & editing. LC: Conceptualization, Funding acquisition, Supervision, Writing – review & editing.

## Supplementary material

SUPPLEMENTARY TABLE S1 All metabolites.

SUPPLEMENTARY TABLE S2 Differential metabolites.

SUPPLEMENTARY TABLE S3 List of KEGG enrichment of differential genes.

SUPPLEMENTARY TABLE S4 All differential genes.
